# Matched vs Nonmatched Placebos in a Randomized Trial of COVID-19 Treatments

**DOI:** 10.1001/jamanetworkopen.2024.10335

**Published:** 2024-05-20

**Authors:** Gilmar Reis, Leonardo Cançado Monteiro Savassi, Thiago Santiago Ferreira, Luiza Lanna França Reis, Maria Izabel Campos Simplicio, Luciene Barra Ribeiro, Eduardo Augusto dos Santos Moreira Silva, Prince Kumar Lat, Ofir Harari, Jamie I. Forrest, Louis Dron, Jay J. H. Park, Kristian Thorlund, Edward J. Mills

**Affiliations:** 1 Research Division, Cardresearch-Cardiologia Assistencial e de Pesquisa, Belo Horizonte, Brazil; 2 Department of Medicine, Pontifícia Universidade Católica de Minas Gerais, Belo Horizonte, Brazil; 3 Department of Health Research Methods, Evidence, and Impact, McMaster University, Hamilton, Ontario, Canada; 4 David Sackett Research Institute, Belo Horizonte, Brazil; 5 Public Health, Mental and Family Medicine Department, Ouro Preto Federal University, Ouro Preto, Brazil; 6 Purpose Life Sciences, Vancouver, British Columbia, Canada; 7 Core Clinical Sciences, Vancouver, British Columbia, Canada; 8 Cascade Outcomes Research, Vancouver, British Columbia, Canada

## Abstract

**Importance:**

Matched placebo interventions are complex and resource intensive. Recent evidence suggests matched placebos may not always be necessary. Previous studies have predominantly evaluated potential bias of nonmatched placebos (ie, differing on dose, frequency of administration, or formulation) in pain and mental health, but to date no systematic examination has been conducted in infectious disease.

**Objective:**

To test for differences between nonmatched and matched placebo arms with respect to clinical outcome measures across multiple therapeutics for COVID-19.

**Design, Setting, and Participants:**

In a comparative effectiveness research study, a post hoc analysis was conducted of data on individual patients enrolled in a large, multiarm, platform randomized clinical trial in symptomatic adult outpatients with COVID-19 between January 15, 2021, to September 28, 2023, in which the outcomes of both matched and nonmatched placebo groups were reported. Bayesian and frequentist covariate-adjusted techniques were compared with 7 intervention-placebo pairs.

**Exposures:**

Seven matched and nonmatched placebo pairs (for a total of 7 comparisons) were evaluated throughout the primary platform trial. Comparisons were made between treatment and its associated matched (concurrent) placebo, as well as with nonmatched placebo (alone and in combination) assessed at a similar time point.

**Main Outcomes and Measures:**

Outcomes assessed included hospitalizations, EuroQol 5-Dimension 5-level scores, and PROMIS Global-10 scores.

**Results:**

A total of 7 intervention-control pairs (N = 2684) were assessed, including 1620 (60.4%) women, with mean (SD) age, 47 (15.2) years; the most common comorbidities were obesity (41.9%) and hypertension (37.9%). In a meta-analysis with decoupled SEs, accounting for overlapping placebo patients, the overall odds ratio (OR) of nonmatched compared with matched placebo was 1.01 (95% credible interval, 0.77-1.32), with posterior probability of equivalence, defined as 0.8 ≤ OR ≤ 1.2 (a deviation from perfect equivalence ie, OR = 1, by no more than 0.2) of 85.4%, implying no significant difference. Unadjusted analysis of the event rate difference between all nonmatched and matched placebo groups did not identify any notable differences across all 7 treatment-placebo combinations assessed. Similar analysis that was conducted for patient-reported quality of life outcomes did not yield statistically significant differences.

**Conclusions and Relevance:**

In this post hoc study of a randomized clinical platform trial, pooling matched and nonmatched placebo patient data did not lead to inconsistencies in treatment effect estimation for any of the investigational drugs. These findings may have significant implications for future platform trials, as the use of nonmatched placebo may improve statistical power, or reduce barriers to placebo implementation.

## Key Points

**Question:**

In COVID-19 randomized clinical trials, is use of nonmatched placebo groups associated with bias in comparative efficacy results?

**Findings:**

In this comparative effectiveness research including 2684 patients across 7 treatments in a single randomized clinical trial, no significant difference between matched and nonmatched placebo populations for comparative effectiveness was observed in both adjusted and unadjusted scenarios.

**Meaning:**

The findings of this study suggest that future studies of a similar design and therapeutic context may not necessitate matched placebo analysis only.

## Teaser

This comparative effectiveness study examines data from randomized clinical trials on use of nonmatched vs matched placebos in treatment of COVID-19.

## Introduction

Randomized clinical trials (RCTs) are essential for establishing evidence of comparative efficacy since prognostic factors (known or unknown) are more equally distributed across treatment groups after randomization compared with nonrandomized populations. Placebo-controlled RCTs in which the placebo intervention has been matched to the characteristics of the experimental intervention (eg, route of administration, frequency, and visual appearance) are considered the standard.^1^

Matched placebo, however, can be complex and time consuming to manufacture, thereby causing delays or added resource requirements for the conduct of RCTs. There are also many clinical settings where the ethics of administering matched placebos may be questioned due to patient discomfort or risks associated with the administration (eg, intravenous infusion).

Several studies have explored the potential bias of matched vs unmatched placebo, but their results have differed. In various areas of pain management (eg, postsurgical or chronic migraine) invasive placebo, such as sham surgery, acupuncture, and subcutaneous injection, have generally been found to yield different responses than oral placebo.^2,3,4,5^ In mental health, meta-analyses of multiple studies suggested that study design and population were predictors of placebo response, rather than the intervention being matched.^3^ Meta-epidemiologic studies across multiple diseases have also found inconsistencies, with close to half of the studies suggesting no bias associated with nonmatched placebo.^6^

Infectious disease RCTs, like many other RCTs, face increasingly challenging resource and ethics constraints. Thus, any substantial improvement in trial conduct efficiencies would help ensure the continued production of high-quality RCTs globally. Additionally, in a rapidly evolving health crisis, such as COVID-19, finding an appropriate matched placebo in a timely manner may not be possible. To our knowledge, no study has previously examined the role of matched vs nonmatched placebo in infectious disease RCTs. Arguably, one of the richest and most valid sources of data for answering such questions is perpetual multiarm trials in which several matched and nonmatched placebo groups have been tested against several experimental interventions within one overarching trial protocol.

The TOGETHER trial was a COVID-19 multiarm trial that assessed 7 interventions including a total of 2684 patients randomized to matching and nonmatching placebo controls.^7^ It explored the effect and differences on hospitalization among COVID-19 outpatients. In this post hoc study, we evaluate comparative effects (or lack thereof) obtained from matched and nonmatched placebo groups on the trial primary outcomes.

## Methods

### Trial Overview and Placebo Matching Details

Our analysis as conducted between January 15, 2021, and September 28, 2023, on patient-level data from the TOGETHER trial. The TOGETHER Trial is a multiarm, randomized platform clinical trial designed to assess the effectiveness of multiple repurposed treatments for COVID-19 among patients who were at risk of developing severe illness. Full details of the TOGETHER trial have been reported previously. In total, the number of patients recruited to the TOGETHER trial exceeds 8000 patients.^8,9,10,11^ Briefly, the primary objective was to investigate potential repurposed interventions to determine whether they can lower the rates of COVID-19 disease progression within 28 days of randomization and up to 60 days postrandomization. Ethical approval followed the CEP-CONEP approval process. Certification of Brazilian ethics approvals were submitted to the Hamilton Integrated Research Ethics Board at McMaster University for final review and approval with informed consent obtained from the participants. The present study was included in the existing TOGETHER trial protocol.

Patients were randomized to individual arms and placebo assignment was stratified to account for other arms in the trial, clinical site, and age (≥50 vs <50 years). Given the multiarm nature of the trial, every active intervention had a matching number of days of placebo, proportionate to the number of active arms in the trial at any given time. Patients assigned to the placebo arm received inert therapy matched to the administration mechanism of the interventional product and for the duration of the interventional product. For example, when looking at the fluvoxamine treatment, we used the matched placebo 10-day dosing as the matched placebo group, and a proportion of the other nonmatched placebos, such as placebo 1-day dosing, Placebo 3-day dosing and placebo 14-day dosing were treated as the nonmatched placebo group. Accordingly, an individual patient may represent a matched placebo comparator for multiple treatments should the placebo regimen overlap with the treatment being evaluated. For example, a patient who received 10 days of oral placebo may be a matched control for both fluvoxamine and metformin comparisons, as they overlap both in time and frequency and administration route.

Specifically, the treatment-placebo arms assessed within this study were fluvoxamine twice daily for 10 days with matched oral placebo for 10 days, fluvoxamine plus budesonide twice daily for 10 days with matched oral and inhaled placebo, pegylated interferon lambda once with matched 1-time subcutaneous placebo, ivermectin once daily for 3 days with matched oral placebo for 3 days, metformin twice daily for 10 days with matched oral placebo for 10 days, famotidine 3 times daily for 10 days with matched oral placebo for 10 days, and spirulina twice daily for 14 days with matched oral placebo for 14 days.

For the nonmatched placebo, we evaluated all placebo-assigned participants outside of the matched placebo population who would have been eligible chronologically at the time that the intervention pairing was assessed to minimize confounding from temporal bias. This is also consistent with the analytical strategy conducted within the original trial reports. An overview of the treatment arms and their associated sample size is presented in eTable 1 in Supplement 1.

### Statistical Analysis

#### Hospitalization Event Rate Calculation

This section outlines the methods used to calculate and compare the event rate, with both the matched and nonmatched placebo arms corresponding to 7 different inventions of the trial. For each analysis corresponding to each intervention assessed, we had 2 groups: nonmatched placebo and matched placebo. The primary outcome event was hospitalization, defined as retention in a COVID-19 emergency setting visits with participants remaining under observation for more than 6 hours or referral to tertiary hospital care for COVID-19 within 28 days of randomization.

We estimated the event rate for placebos in each treatment arm without any covariate adjustment. We used the binomial distribution to model these events, considering the number of patients and the event rate. We assigned the individual event rates independent uniform distributions on the unit interval, leading to β posterior distributions. Inference on the event rate differences was conducted using four 20 000-long Monte Carlo samples from said posteriors, with the first half being discarded. In addition to the comparison of event rates between the individual nonmatched placebos and the corresponding matched placebo, nonmatched placebos were also pooled and were compared with the matched placebo group for each treatment arm.

#### Covariate-Adjusted Odds Ratio Calculation

Additionally, as the observed differences between placebo groups could be attributable to nonplacebo treatment-related characteristics, namely age group (≥50 vs <50 years), sex, and body mass index (BMI) (≥30 vs <30, calculated as weight in kilograms divided by height in meters squared), we used bayesian logistic regression to estimate the odds ratio (OR) of each nonmatched placebo compared with the respective matched placebo group. Given that 98% of the participants identified as mixed race, insufficient numbers of patients were evaluable to consider the use of race and ethnicity within our model. The brms package in R (R Foundation for Statistical Computing)^12^ was used for this purpose, running 20 000 iterations spread across 4 chains. Point and interval estimation was based on the resultant posterior samples. As in the event rate calculation, besides estimating individual pairwise ORs for each nonmatched placebo compared with the matched placebo, we also grouped nonmatched placebos within a treatment arm and compared them with the corresponding matched placebo group of the same arm.

#### Pooled OR via Decoupled Study Meta-Analysis

To get a sense of an overall nonmatched vs matched placebo effect, we fitted logistic regressions models of hospitalization on age, sex, and BMI as before, and recorded the estimated ORs and SEs. Most of these estimates were based on overlapping sets of patients and were therefore dependent. We thus applied the decoupling approach of Han et al^13^ to yield independent SEs before conducting meta-analysis.

#### Patient-Reported Outcomes

Acknowledging that there may be a differential impact of outcome types with respect to between-placebo differences, we also explored 2 patient-reported outcomes. In the substudies involving fluvoxamine, fluvoxamine plus budesonide, ivermectin, pegylated interferon lambda, and metformin, we assessed the PROMIS Global-10 score, a 10-item questionnaire whose items are all 5-point individual T scores gauging health care–related quality of life^14^ and the EuroQol 5-Dimension 5-level (EQ-5D-5L) questionnaire, consisting of five 5-level items, was conducted, covering mobility, self-care, usual activities, pain/discomfort, and anxiety/depression. These questionnaires were conducted on multiple occasions throughout the trial, and we analyzed the day 28 results of both.

We analyzed the selected outcomes for each study (ie, each treatment with its associated placebo comparison), fitting a multivariate linear regression model for the PROMIS Global-10 scores (obtained by summing the individual scores). The EQ-5D-5L dimensions were converted to a numeric score in the 0 to 1 range, using the valuation technology value set using the eq5d R library.^15^ More than 50% of the patients had the maximum score of 1 on day 28, and we used Tobit regression for truncated data to address it,^16^ using the MCMCpack R library.^17^ Analysis for both outcomes was conducted with adjustment for age, sex, and BMI, as well as the baseline score. Because data were missing for approximately 15% of the patients, we performed multivariate imputations with chained equations using predictive mean matching with 15 imputed data sets.^18^

## Results

**Figure 1.**
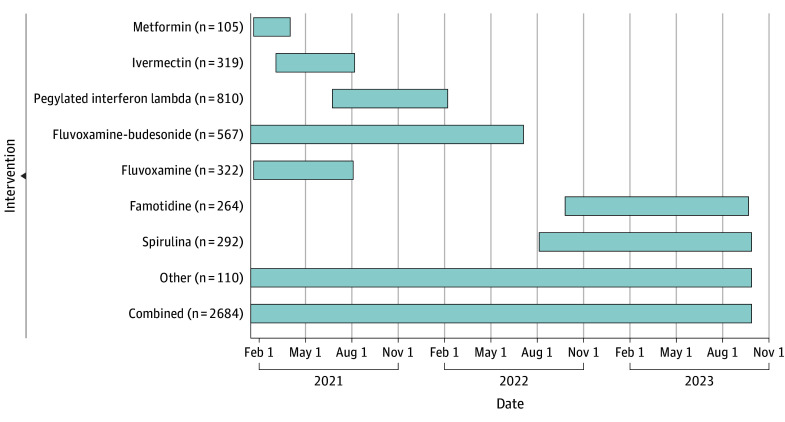
Outcomes in Patients Receiving Placebo Some placebo regimens match more than 1 treatment, such as the 10-day dosing that matches both metformin and fluvoxamine. Numbers do not include 3 patients shared between the fluvoxamine, ivermectin, and metformin placebo arms whose regimen was not recorded.

We included a total of 2684 placebo-receiving patients from 7 different treatment arm pairs. Among these, 1620 (60.4%) were females and 1063 (39.6%) were males (data missing on 1 patient). Additionally, 52.2% of these patients were younger than 50 years, with mean (SD) age, 47 (15.2) years. The timeline and numbers of the different placebo doses that form the analysis set presented herein are displayed in Figure 1. Further details on the included placebo population are provided in eTable 1 and eTable 2 in Supplement 1.

The mean (SD) sample size per comparison (ie, the total matched and nonmatched placebo participants for each comparison) was 638 (247), with the lowest number of evaluable patients in metformin (n = 202) arm and the highest number in the pegylated interferon lambda arm (n = 1003). These differences are attributable both to the time at which the intervention pairing was assessed and the eligible nonmatched placebo population for each such comparison. The breakdown for the numbers of matched vs nonmatched placebo for each study has been summarized in eTable 1 in Supplement 1.

For unadjusted analysis of the primary outcome of hospitalization, we found no notable difference in the event rate between the matched and the nonmatched placebo groups across all treatments assessed (Table 1). Additional comparisons with a breakdown per each nonmatched control are available in eTable 3 in Supplement 1. Furthermore, we did not find any substantial difference in the risk of an event occurring in either the individual or the combined nonmatched placebo arm compared with the matched placebo arm of the treatment arms after adjusting for age, sex, and BMI. The bar plot showing the event rate comparison and the density plot showing the posterior distributions of ORs for each comparison are presented in eFigure 1 and eFigure 2 in Supplement 1.

**Table 1.** Event Rate and OR of Matched vs Nonmatched Placebo for Hospitalizations Across Different Treatment Arms

Arm	Patients, No.	Unadjusted event (95% CrI)	Covariate AOR (95% CrI)^a^	Bayesian posterior probability of equivalence, %^b^
Metformin				
Matched placebo 10 d	105	17.8 (11.2-25.5)	NA	NA
Nonmatched placebo combined	97	15.2 (8.8-22.8)	0.67 (0.29-1.52)	25.7
Ivermectin				
Matched placebo 3 d	319	16.5 (12.7-20.8)	NA	NA
Nonmatched placebo combined	358	16.4 (12.8-20.4)	1.08 (0.70-1.65)	60.4
Pegylated interferon lambda				
Matched placebo 1 d	810	5.4 (4.0-7.1)	NA	NA
Nonmatched placebo combined	193	8.2 (4.8-12.4)	1.15 (0.60-2.10)	42.1
Fluvoxamine-Budesonide				
Matched placebo 10 d	567	3.9 (2.4-5.6)	NA	NA
Nonmatched placebo combined	171	4.0 (1.7-7.4)	0.97 (0.34-2.37)	32.7
Fluvoxamine				
Matched placebo 10 d	322	16.4 (12.5-20.6)	NA	NA
Nonmatched placebo combined	431	15.5 (12.2-19.0)	0.92 (0.61-1.39)	64.8
Famotidine				
Matched placebo 10 d	264	1.5 (0.4-3.3)	NA	NA
Nonmatched placebo combined	274	1.4 (0.4-3.2)	0.96 (0.17-5.30)	19.3
Spirulina				
Matched placebo 14 d	292	1.7 (0.6-3.5)	NA	NA
Nonmatched placebo combined	265	1.5 (0.4-3.3)	0.81 (0.16-3.79)	20.0

Abbreviations: AOR, adjusted odds ratio; CrI, credible interval; NA, not applicable; OR, odds ratio.

^a^ Odds ratio of nonmatched vs matched placebo adjusted with respect to age, sex, and body mass index.

^b^ Equivalence is defined as Pr |(OR − 1|≤0.2|data).

**Figure 2.**
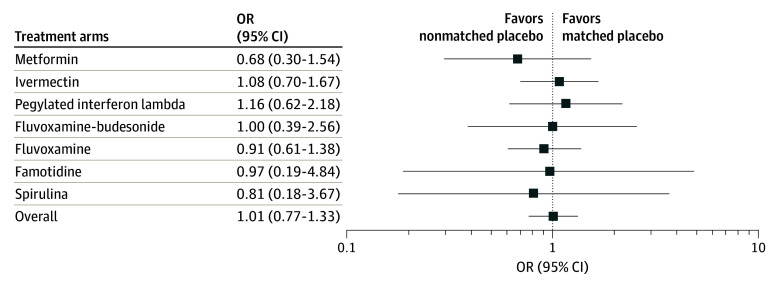
Covariate-Adjusted Odds Ratio (OR) for Each Study

To estimate a pooled effect, for a more comprehensive assessment of the difference between the nonmatched and matched placebo, we accrued data from all 7 comparisons of the primary outcome. First, we derived estimates for the log OR (and their associated SEs) from all studies, while adjusting for sex, age, and BMI. We then applied the decoupling approach of Han et al^13^ to obtain independent SEs and performed meta-analysis on the resultant study level effects. Figure 2 summarizes the findings. The combined overall effect estimation indicates that there is no discernible difference in the event rate between the nonmatched and matched placebo groups (OR, 1.01; 95% CI, 0.77-1.33), suggesting that the occurrence of hospitalizations is comparable for both types of placebos across the entire study population. No study heterogeneity was detected (*I*^2^ = 0.0%); hence, the fixed and the random effect models retrieved identical results.

**Figure 3.**
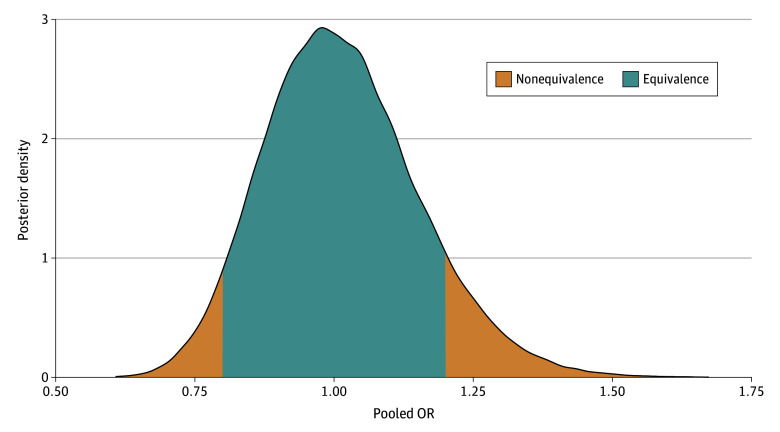
Probability Density Function for the Posterior Distribution of the Pooled OR of the Nonmatched Placebo Relative to Matched Placebo for the Composite Outcome of Hospitalization and Extended Emergency Visit The equivalence between the 2 groups was defined as 0.8≤OR≤1.2.

Furthermore, we fitted a bayesian meta-analysis to calculate a posterior probability of equivalence between the placebo groups, using an ad hoc definition of probability of equivalence (0.8≤OR≤1.2 data). This resulted in an identical OR estimate of 1.01, with a virtually identical uncertainty interval (95% credible interval, 0.77-1.32). More importantly, the posterior probability of equivalence of the pooled OR was 85.4%, contrasting with the arm-specific probabilities that ranged between 19% (famotidine) and 65% (fluvoxamine) as presented in Table 1. Figure 3 shows the posterior OR probability density function.

To also address subjective outcomes, we analyzed the EQ-5D-5L results, as well as the PROMIS Global-10 results for the remaining studies. Table 2 contains the results of a linear model for the PROMIS Global-10 outcome and a Tobit regression for the EQ-5D-5L outcome, both after adjustment for age, sex, BMI, and baseline score, where neither end point showed any important difference between the matched and the nonmatched placebo.

**Table 2.** Mean Difference of Matched vs Nonmatched Placebo for Patient-Reported Outcomes Across Different Treatment Arms

Matched vs nonmatched placebo arm^a^	Covariate-adjusted mean difference (95% CrI)
Metformin	−0.40 (−1.79 to 0.98)
Ivermectin	−0.31 (−1.21 to 0.59)
Pegylated interferon lambda	0.82 (−0.04 to 1.67)
Fluvoxamine plus budesonide	−0.10 (−1.06 to 0.86)
Fluvoxamine	0.03 (−0.81 to 0.87)
Famotidine	−0.02 (−0.06 to 0.01)
Spirulina	0.02 (−0.02 to 0.06)

Abbreviation: CrI, credible interval.

^a^ Spirulina and famotidine were assessed with the EuroQol 5-Dimension 5-level score, whereas other therapies were assessed with the PROMIS Global-10 score.

## Discussion

In this reanalysis of a prospectively enrolling platform randomized clinical trial, we were able to compare patients who received matched placebo interventions with those assigned to various placebo arms in different substudies and have observed that the probability of equivalent outcomes between matched placebo and unmatched placebo was 85.4%.

These data suggest that in the circumstances observed with respect to clinical context and outcomes assessed, placebo arms may not necessarily need to match experimental interventions; shared or borrowed placebo groups with different dosing schedules may be a viable substitute. These choices are of particular importance for subsequent studies wherein the choice of nonmatched placebos, including shared or borrowed placebo groups with different dosing schedules, may reduce patient burden through mimicking burdensome treatment regimens or exposure to supportive therapies unnecessary in the placebo-receiving population.

To our knowledge, this study is the first direct comparison exploring potential variations among multiple different placebo administration and duration varieties within a single randomized clinical trial. Using data from a single clinical trial, our investigation stands in contrast to prior efforts that either indirectly approached the question through meta-analysis of separate trials comparing placebo group changes^2,3,19,20^ or selected randomized clinical trials with explicit randomization to distinct placebo groups.^6,21^

The results of prior studies have been heterogeneous and associated with variability in interpretation. For instance, Meissner et al^19^ used a meta-analysis to report that sham acupuncture and sham surgery exhibited higher responder ratios than oral pharmacologic placebo arms in treating patients with migraine. Additional inquiries^5,21,22,23^ reached similar conclusions, varying in the certainty of placebo effects linked to a multitude of medical practices. Conversely, several studies^24,25,26,27,28,29,30^ have negated the presence of differences when using various placebo strategies within a treatment arm during clinical trials. These disparities can be attributed to limitations such as publication bias, interstudy heterogeneity, contextual specificity, study selection bias, and time lag. In contrast, the present study provides an analysis population captured under a singular master protocol umbrella, improving the ability to compare patient populations. Furthermore, we used population-adjustment analyses to evaluate the impact of potential between-group differences, which did not alter the conclusions of this work.

### Limitations

Our study has some limitations with respect to its generalizability. Our analysis was restricted to a single trial and 3 outcomes. Accordingly, whether our findings are reflective of the unique circumstances of the analyzed trial, such as its geography, patient population, or other operational characteristics, cannot be examined in the present analysis. However, to our knowledge, the assessed trial was the largest placebo-controlled trial conducted during the COVID-19 pandemic and enrolled more than 8000 patients. Several comparisons are hindered by small sample sizes. In particular, several nonmatched placebo comparisons in eTable 3 in Supplement 1 are limited by sample sizes of less than 50 and are associated with large credible intervals. Accordingly, the comparative estimates in our covariate-adjusted analyses are particularly wide. Therefore, while the pooled nonmatched placebo estimates remain comparatively robust, our current data are insufficient for all pairwise comparisons that may be of interest. Furthermore, while our study is contained within 1 protocol, substantial meta-effects may be at play given the differences in time of measurement of outcomes. We observed substantial changes with respect to base event rates between placebo-treated populations over time, generally decreasing in event rate. These temporal factors are important to consider in the context of contemporary COVID-19 studies, as the interplay between vaccination coverage, medical care, population-level exposure, and variants may influence our findings.

## Conclusions

The results of this study suggest that, when analyzed in a single trial evaluating multiple treatment and placebo comparisons, the choice of matched vs nonmatched controls is not associated with inferential changes with respect to subjective or objective outcomes among outpatients with COVID-19. Our data set, consisting of a single multiarm trial with multiple placebo arms, is unique in its ability to assess the choice of both matched and nonmatched placebo, without being subject to the between-study differences associated with other meta-analyses. The implication of this finding is particularly important for study designs evaluating multiple treatments where it is not possible to match placebo, providing evidence that a nonmatched alternative may not have important effects on results.

## Notes

Supplement 1. **eFigure 1.** Event Rate Across All Multiple Studies in the TOGETHER Trial **eFigure 2.** Posterior Distribution of Odds Ratio (OR) for Non-matched vs Matched Placebo in Different Studies **eTable 1.** An Overview of Treatments, Matched and Non-matched Placebo Treatments Assessed in This Analysis **eTable 2.** A Summary of Baseline Characteristics (Matched Placebo Populations) **eTable 3.** A Complete Overview of Matched and Non-matched Placebo for Hospitalizations Across Different Treatment Arms

Supplement 2.
Data Sharing Statement
