# Supplementary material for: Matched vs Nonmatched Placebos in a Randomized Trial of COVID-19 Treatments
Source: JAMA Netw Open. 2024 May 20;7(5):e2410335. doi: 10.1001/jamanetworkopen.2024.10335 (PMC11107303; doi:10.1001/jamanetworkopen.2024.10335)
Supplement: Supplement 1. — eFigure 1. Event Rate Across All Multiple Studies in the TOGETHER Trial eFigure 2. Posterior Distribution of Odds Ratio (OR) for Non-matched vs Matched Placebo in Different Studies eTable 1. An Overview of Treatments, Matched and Non-matched Placebo Treatments Assessed in This Analysis eTable 2. A Summary of Baseline Characteristics (Matched Placebo Populations) eTable 3. A Complete Overview of Matched and Non-matched Placebo for Hospitalizations Across Different Treatment Arms [file jamanetwopen-e2410335-s001.pdf]

Supplementary Online Content

Reis G, Savassi LCM, Ferreira TS, et al. Matched vs nonmatched placebos in a randomized trial of COVID-19 treatments. *JAMA Netw Open*. 2024;7(5):e2410335. doi:10.1001/jamanetworkopen.2024.10335

eFigure 1. Event Rate Across All Multiple Studies in the TOGETHER Trial

eFigure 2. Posterior Distribution of Odds Ratio (OR) for Non-matched vs Matched Placebo in Different Studies

eTable 1. An Overview of Treatments, Matched and Non-matched Placebo Treatments Assessed in This Analysis

eTable 2. A Summary of Baseline Characteristics (Matched Placebo Populations)

eTable 3. A Complete Overview of Matched and Non-matched Placebo for Hospitalizations Across Different Treatment Arms

This supplementary material has been provided by the authors to give readers additional information about their work.

© 2024 Reis G et al. *JAMA Network Open.*

Supplementary data for: Are matched placebos important in randomized trials of infectious diseases?

Supplementary Data

a.)

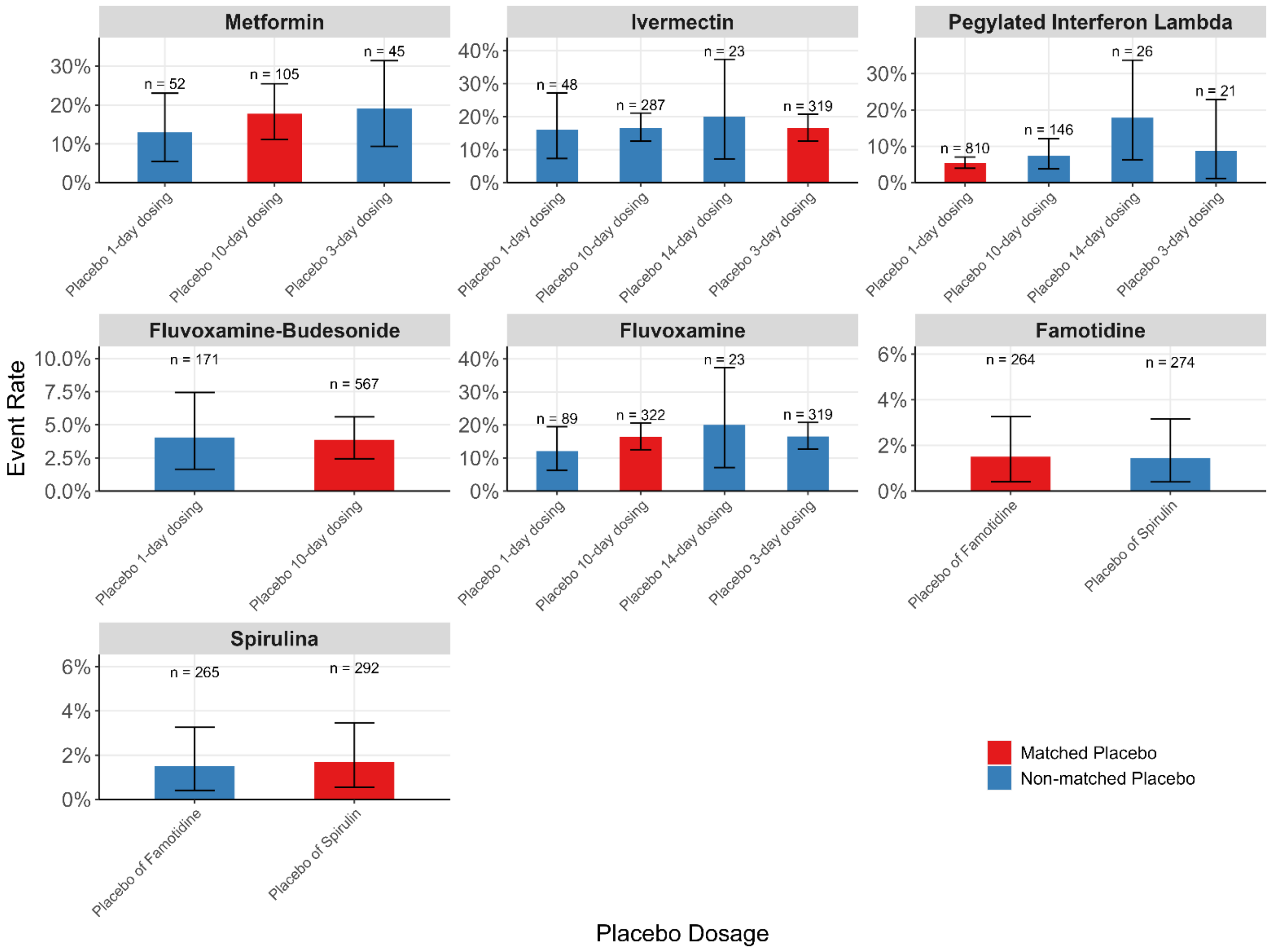

© 2024 Reis G et al. *JAMA Network Open.*

b.)

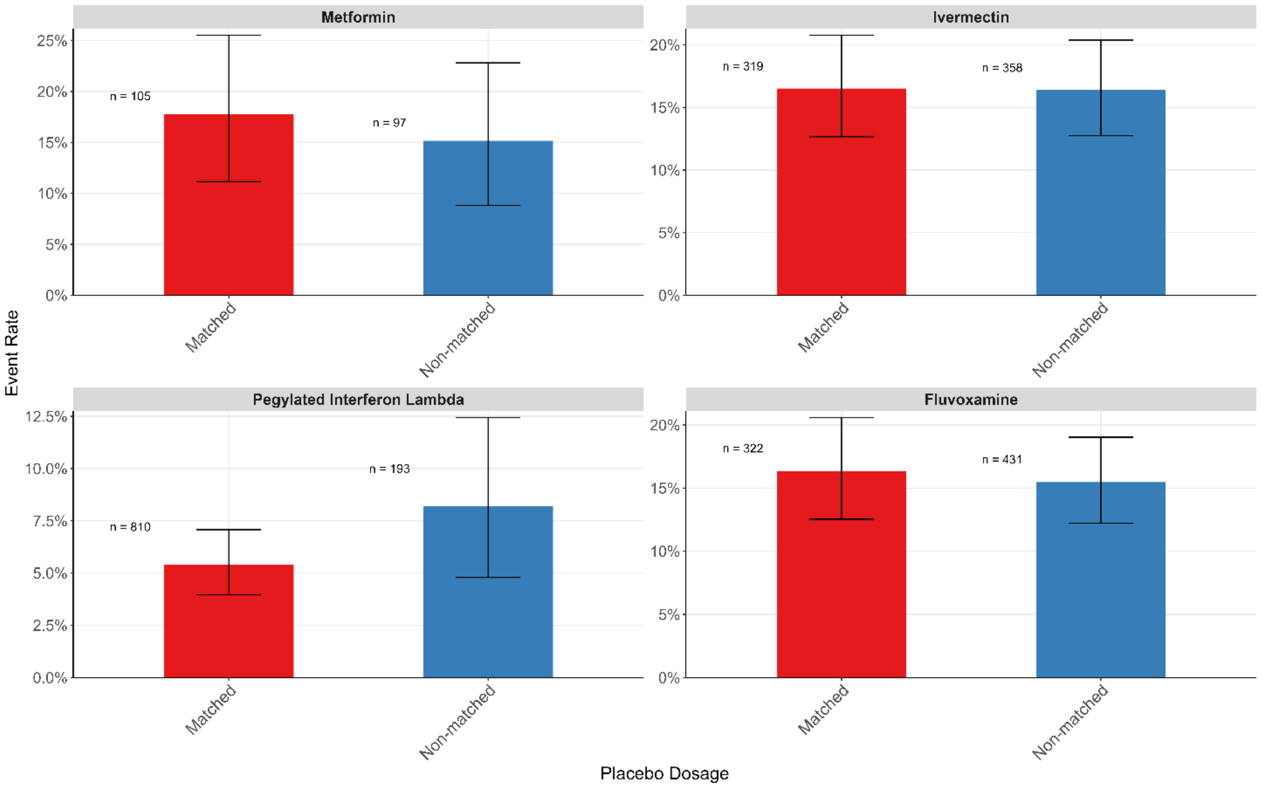

Supplementary Figure 1. Event rate across all multiple studies in the TOGETHER trial. a.) event rate in individual non-matched and matched placebos for study. b.) event rate in unified non-matched placebos and corresponding matched placebos in studies with more than one non-matched placebo.

© 2024 Reis G et al. *JAMA Network Open.*

Supplementary Figure 2

a) Metformin

i. Individual non-matched dose

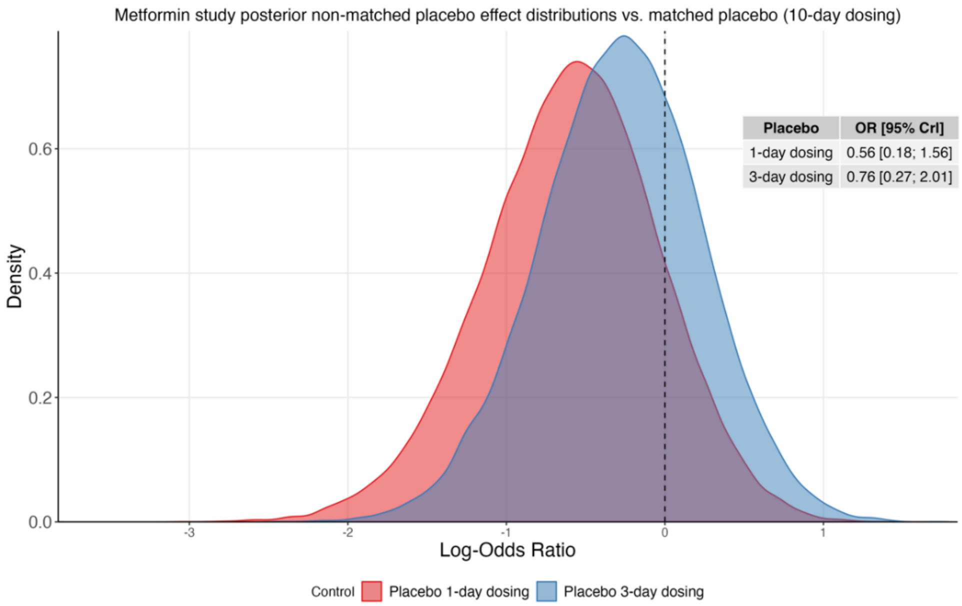

ii. Combined non-matched doses

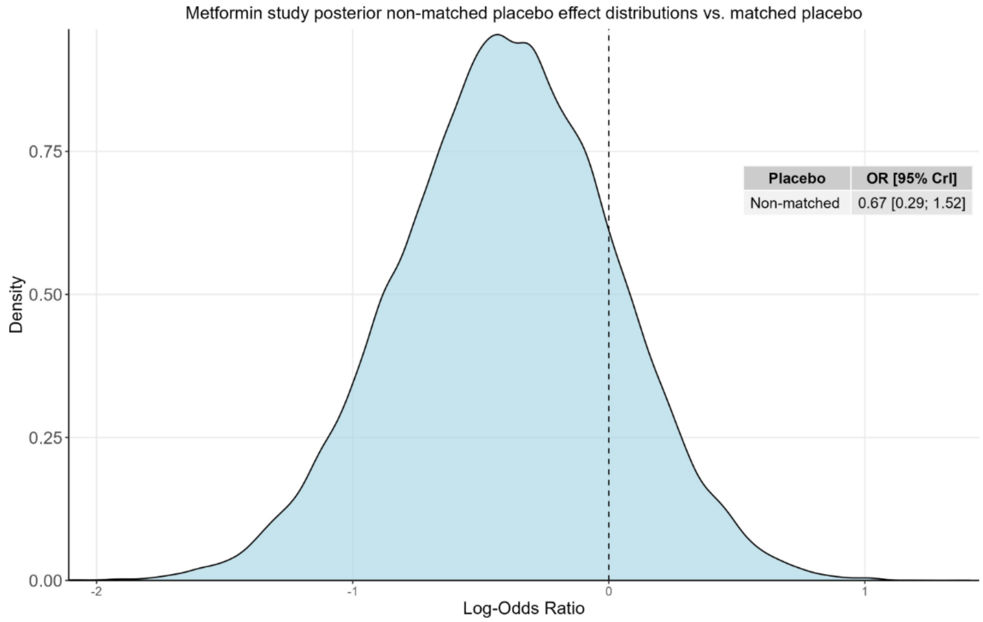

© 2024 Reis G et al. *JAMA Network Open.*

b) Ivermectin

i. Individual non-matched dose

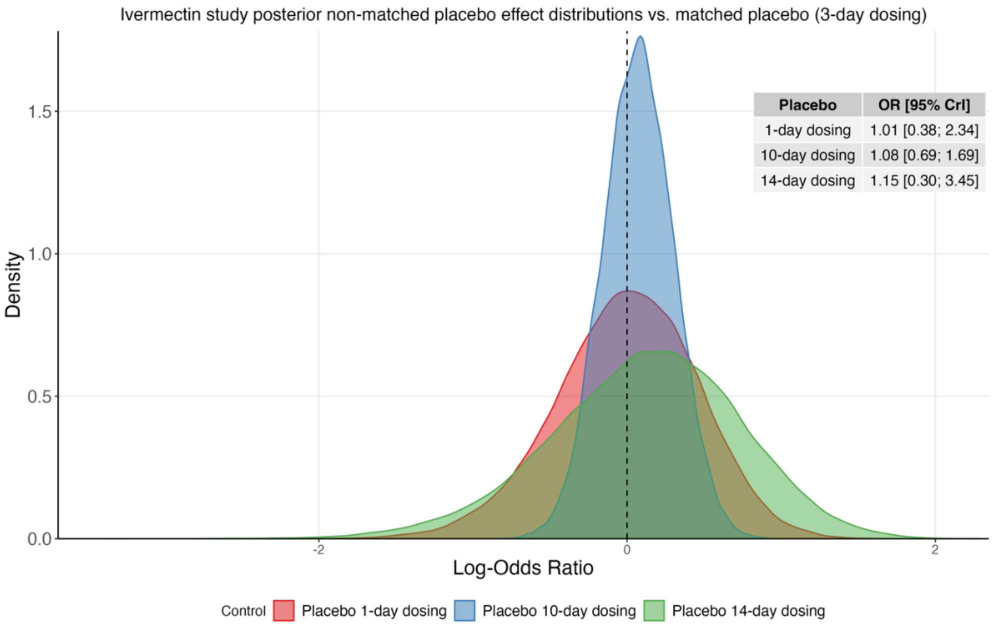

ii. Combined non-matched doses

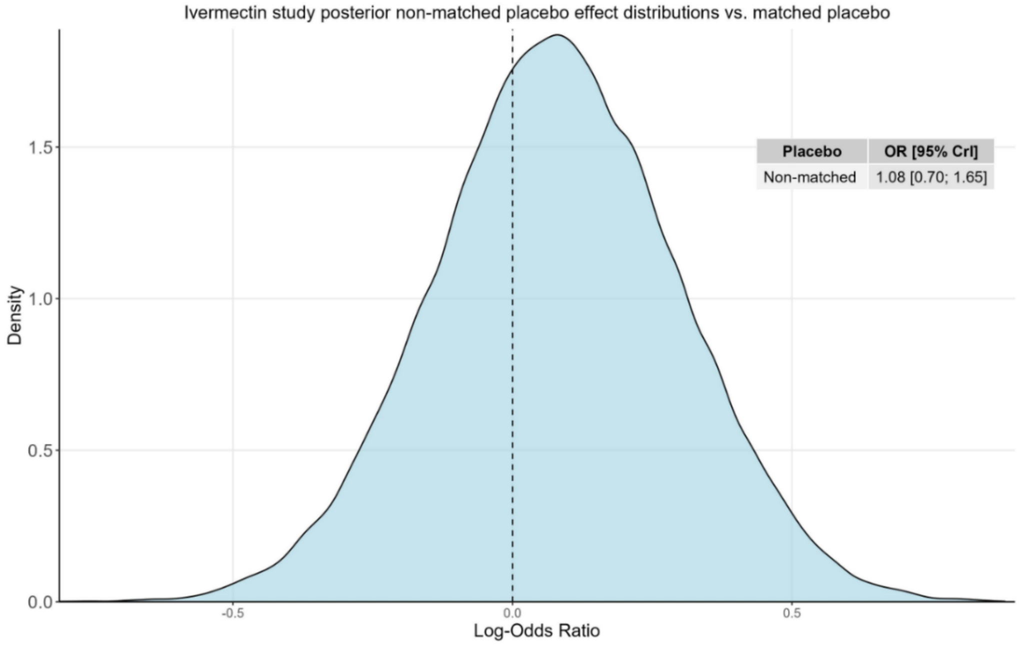

© 2024 Reis G et al. *JAMA Network Open.*

c) Pegylated Interferon Lambda

i. Individual non-matched dose

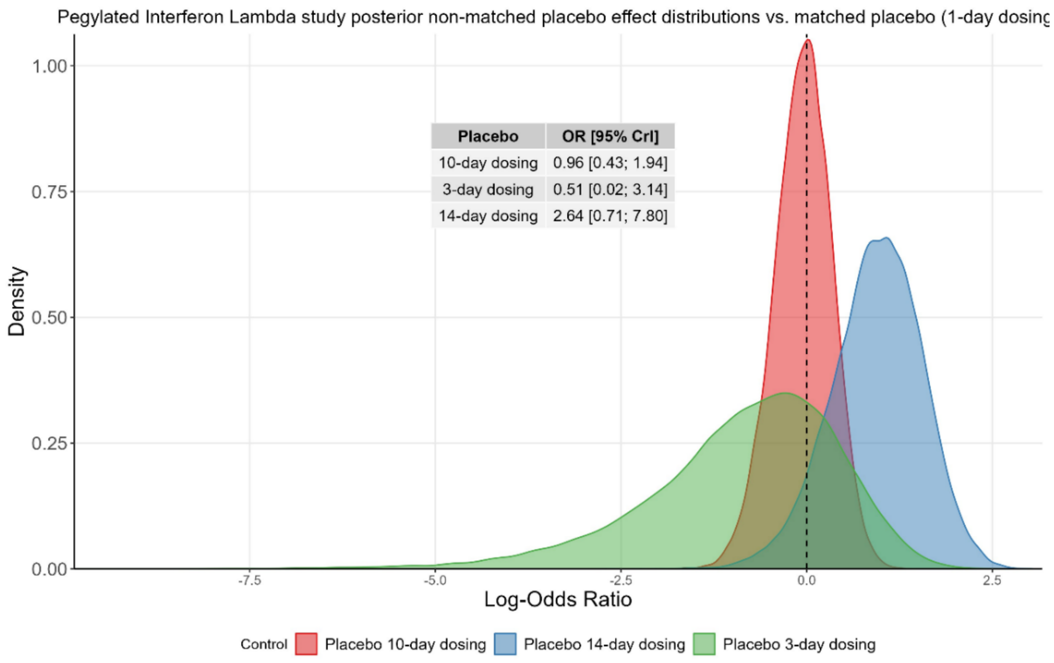

ii. Combined non-matched doses

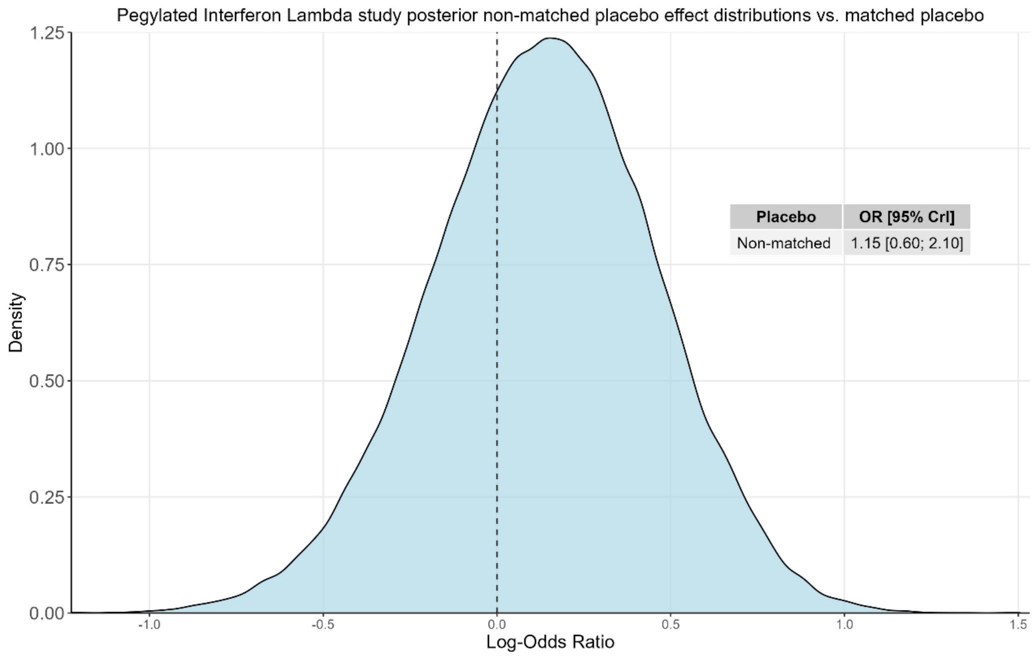

© 2024 Reis G et al. *JAMA Network Open.*

d) Fluvoxamine-Budesonide

i. Combined non-matched doses

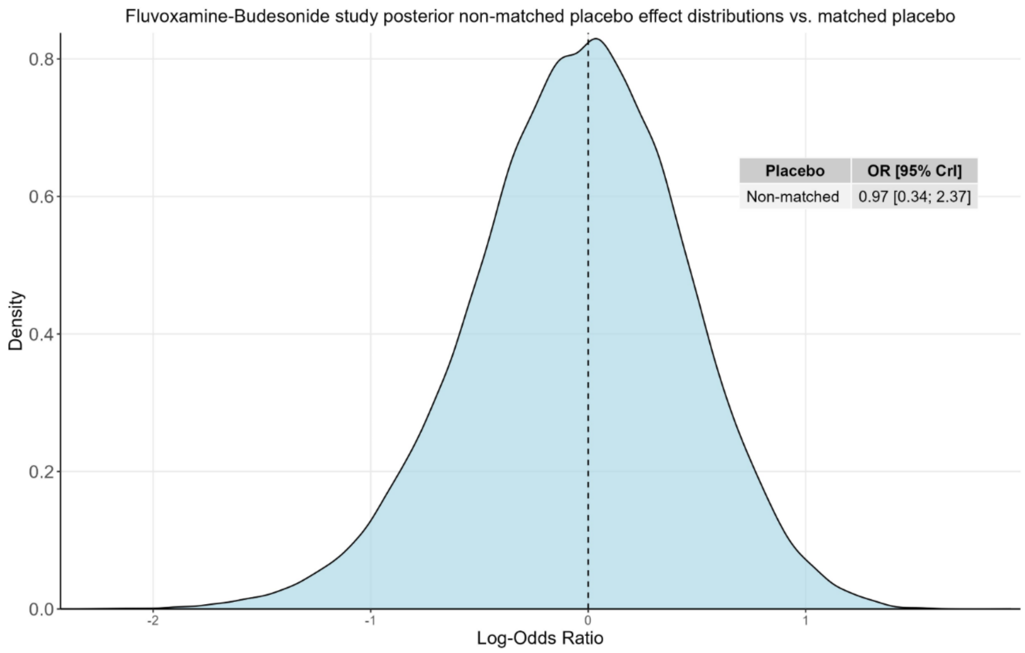

e) Fluvoxamine

i. Individual non-matched dose

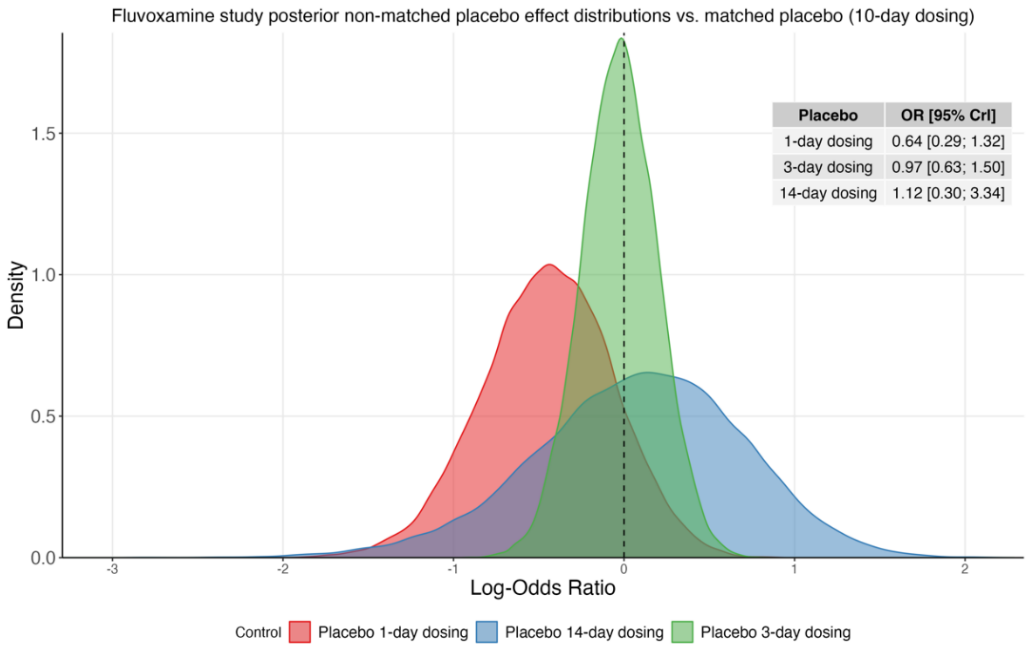

© 2024 Reis G et al. *JAMA Network Open.*

ii. Combined non-matched doses

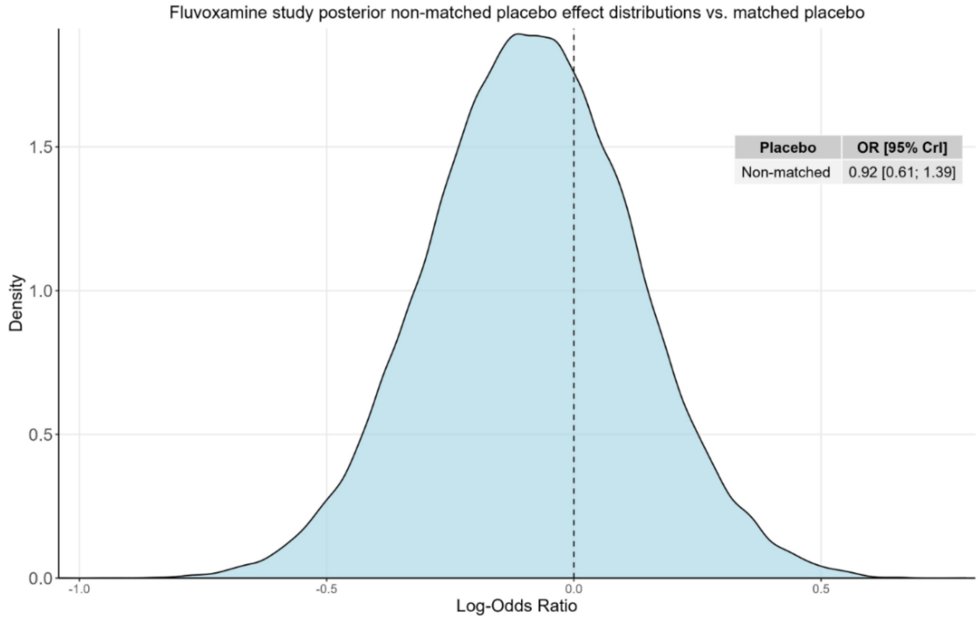

f) Famotidine

i. Combined non-matched doses

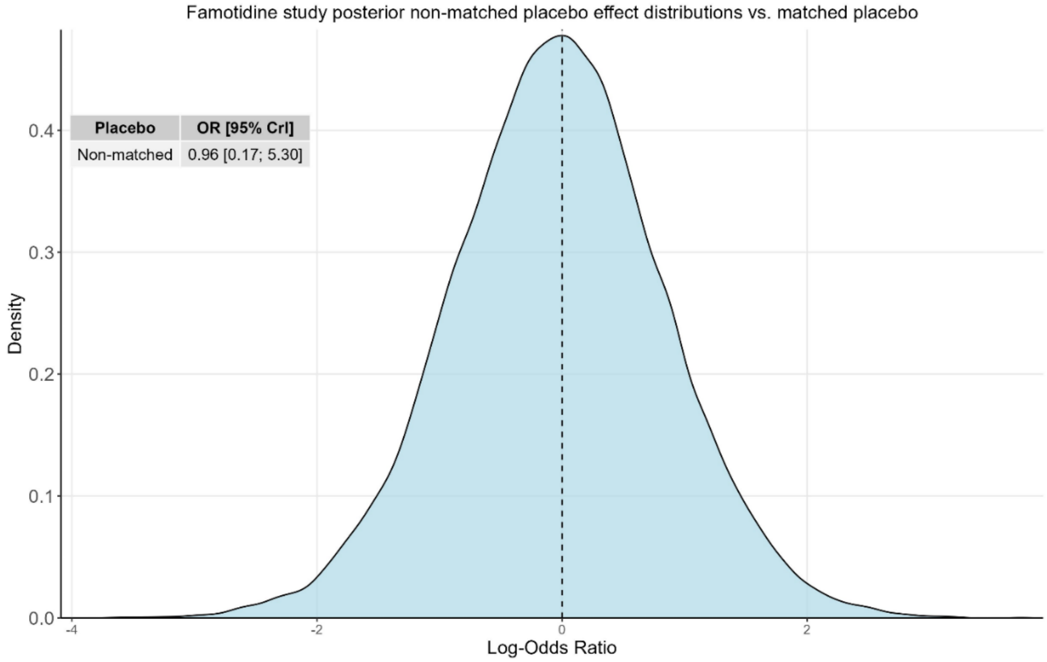

© 2024 Reis G et al. *JAMA Network Open.*

g) Spirulina

i. Combined non-matched doses

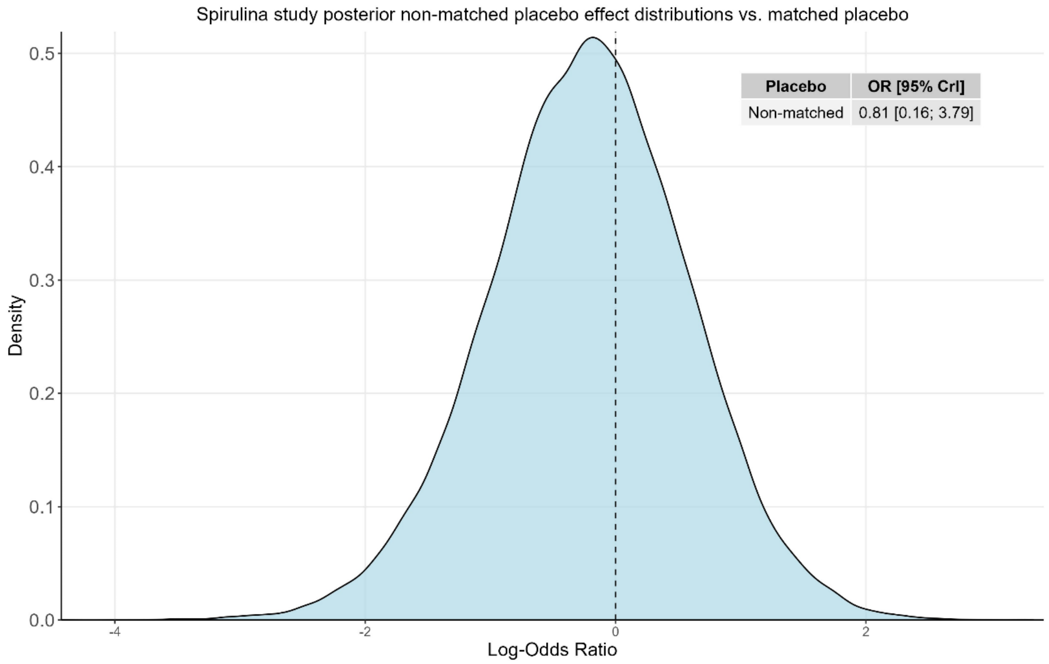

Supplementary Figure 2. Posterior distribution of odds ratio (OR) for non-matched vs matched placebo in different studies. Subplots from (a-g) shows the density plot of OR distribution for the non-matched placebos compared to matched ones. Top panel in (a-d) shown individual non-matched placebo comparison to the matched one and the bottom panel shows the combined non-matched placebo comparison to the matched one. In Fluvoxamine-Budesonide, Spirulina and Famotidine, there was only one non-matched placebo arm.

© 2024 Reis G et al. *JAMA Network Open.*

Supplementary Table 1: An overview of treatments, matched and non-matched placebo treatments assessed in this analysis.

Treatment, administration, frequency	Matched Placebo Type	Matched Placebo N	Non-matched Placebo Type	N
Metformin, 750mg extended-release tablets administered orally twice daily for 10 days	Placebo 10-day (oral)	105	Placebo 1-day	52
			Placebo 3-day	45
			Overall	97
Ivermectin, 400 µg per kilogram of body weight once daily orally for 3 days	Placebo 3-day (oral)	319	Placebo 1-day	48
			Placebo 10-day	287
			Placebo 14-day	23
			Overall	358
Pegylated Interferon Lambda, Subcutaneous injection of 180µg pegylated interferon lambda, (0.45ml) single dose	Placebo 1-day (subcutaneous)	810	Placebo 3-day	21
			Placebo 10-day	146
			Placebo 14-day	26
			Overall	193
Fluvoxamine-Budesonide, 100mg tables administered orally plus inhaled budesonide 800ug twice daily for 10 days	Placebo 10-day (oral and inhaled)	567	Placebo 1-day	171
			Overall	171
Fluvoxamine, 100mg tablets administered orally twice daily for 10 days	Placebo 10-day (oral)	322	Placebo 1-day	89
			Placebo 3-day	319
			Placebo 14-day	23
			Overall	431
Famotidine 80 mg administered orally thrice daily for 10 days	Placebo 10-day (oral)	264	Placebo 14-day	274
			Overall	274
Spirulina 500mg administered orally twice daily for 14 days	Placebo 14-day (oral)	292	Placebo 10-day	265
			Overall	265
Other			Overall	110

Note: "Other" refers to placebo doses given that did not match any concurrent treatment at the time of administration.

© 2024 Reis G et al. *JAMA Network Open.*

Supplementary Table 2: A summary of baseline characteristics (matched placebo populations)

	Famotidine (N=264)	Fluvoxamine (N=322)	Fluvoxamine-Budesonide (N=567)	Pegylated Interferon Lambda (N=810)	Ivermectin (N=319)	Metformin (N=105)	Spirulina (N=292)
**Sex n (%)**							
Female	171 (64.8)	200 (62.1)	346 (61.0)	467 (57.7)	186 (58.3)	66 (62.9)	186 (63.7)
Male	93 (35.2)	122 (37.9)	221 (39.0)	343 (42.3)	133 (41.7)	39 (37.1)	105 (36.0)
Unknown	0 (0)	0 (0)	0 (0)	0 (0)	0 (0)	0 (0)	1 (0.3)
**Race n (%)**							
Mixed Race	261 (98.9)	314 (97.5)	551 (97.2)	787 (97.2)	313 (98.1)	99 (94.3)	289 (99.0)
Unknown	3 (1.1)	3 (0.9)	0 (0)	0 (0)	2 (0.6)	2 (1.9)	3 (1.0)
Black or African American	0 (0)	2 (0.6)	4 (0.7)	8 (1.0)	2 (0.6)	2 (1.9)	0 (0)
White	0 (0)	3 (0.9)	12 (2.1)	15 (1.9)	2 (0.6)	2 (1.9)	0 (0)
**Age n (%)**							
<=50	130 (49.2)	160 (49.7)	241 (42.5)	515 (63.6)	159 (49.8)	39 (37.1)	150 (51.4)
>50	134 (50.8)	162 (50.3)	326 (57.5)	295 (36.4)	160 (50.2)	66 (62.9)	140 (47.9)
Missing	0 (0)	0 (0)	0 (0)	0 (0)	0 (0)	0 (0)	2 (0.7)
**Age**							
Mean (SD)	48.3 (15.2)	48.6 (14.0)	49.9 (15.0)	43.3 (15.3)	47.3 (13.6)	52.8 (16.1)	47.6 (16.2)
Median [Min, Max]	50.0 [18.0, 84.0]	50.0 [18.0, 90.0]	51.0 [18.0, 84.0]	41.0 [18.0, 85.0]	50.0 [19.0, 102]	52.0 [18.0, 90.0]	48.0 [19.0, 92.0]
Missing	1 (0.4)	0 (0)	0 (0)	0 (0)	0 (0)	0 (0)	1 (0.3)
**Hypertension n (%)**	107 (40.5)	131 (40.7)	256 (45.2)	237 (29.3)	136 (42.6)	44 (41.9)	107 (36.6)
**Cardiovascular Disease n (%)**	3 (1.1)	3 (0.9)	23 (4.1)	23 (2.8)	6 (1.9)	1 (1.0)	7 (2.4)
**Lung Disease n (%)**	3 (1.1)	10 (3.1)	15 (2.6)	23 (2.8)	10 (3.1)	2 (1.9)	2 (0.7)
**Asthma n (%)**	26 (9.8)	31 (9.6)	58 (10.2)	81 (10.0)	26 (8.2)	8 (7.6)	19 (6.5)
**Type 1 Diabetes n (%)**	6 (2.3)	5 (1.6)	12 (2.1)	8 (1.0)	4 (1.3)	3 (2.9)	2 (0.7)
**Type 2 Diabetes n (%)**	35 (13.3)	42 (13.0)	101 (17.8)	72 (8.9)	48 (15.0)	11 (10.5)	28 (9.6)
**Obesity n (%)**	105 (39.8)	161 (50.0)	225 (39.7)	312 (38.5)	168 (52.7)	47 (44.8)	105 (36.0)

© 2024 Reis G et al. *JAMA Network Open.*

Supplementary Table 2: A summary of baseline characteristics (matched placebo populations)

	Famotidine (N=264)	Fluvoxamine (N=322)	Fluvoxamine-Budesonide (N=567)	Pegylated Interferon Lambda (N=810)	Ivermectin (N=319)	Metformin (N=105)	Spirulina (N=292)
**Cancer n (%)**	5 (1.9)	7 (2.2)	18 (3.2)	9 (1.1)	1 (0.3)	3 (2.9)	5 (1.7)

© 2024 Reis G et al. *JAMA Network Open.*

Supplementary Table 3: A complete overview of matched and non-matched placebo for hospitalizations across different treatment arms.

**Arm**		**n**	**Unadjusted Event (%)** [95% CrI]	**Covariate Adjusted Odds Ratio** [95% CrI]
Metformin	Matched Placebo 10-day	105	17.8 [11.2; 25.5]	
	Non-matched Placebo 1-day	52	13.0 [5.5; 23.0]	0.56 [0.18 – 1.56]
	Non-matched Placebo 3-day	45	19.1 [9.4; 31.4]	0.76 [0.27 – 2.01]
	Non-matched Placebo combined	97	15.2 [8.8; 22.8]	0.67 [0.29 – 1.52]
Ivermectin	Matched Placebo 3-day	319	16.5 [12.7; 20.8]	
	Non-matched Placebo 1-day	48	16.0 [7.3; 27.2]	1.01 [0.38 – 2.34]
	Non-matched Placebo 10-day	287	16.6 [12.6; 21.1]	1.08 [0.69 – 1.69]
	Non-matched Placebo 14-day	23	20.0 [7.1; 37.4]	1.15 [0.30 – 3.45]
	Non-matched Placebo combined	358	16.4 [12.8; 20.4]	1.08 [0.70 – 1.65]
Pegylated Interferon Lambda	Matched Placebo 1-day	810	5.4 [4.0; 7.1]	
	Non-matched Placebo 3-day	21	8.7 [1.1; 22.8]	0.51 [0.02 – 3.14]
	Non-matched Placebo 10-day	146	7.4 [3.8; 12.2]	0.96 [0.43– 1.94]
	Non-matched Placebo 14-day	26	17.9 [6.3; 33.7]	2.64 [0.71 – 7.80]
	Non-matched Placebo combined	193	8.2 [4.8; 12.4]	1.15 [0.60 – 2.10]
Fluvoxamine-Budesonide	Matched Placebo 10-day	567	3.9 [2.4; 5.6]	

© 2024 Reis G et al. *JAMA Network Open.*

	Non-matched Placebo 1-day	171	4.0 [1.7; 7.4]	0.97 [0.34 – 2.37]
	Non-matched Placebo combined	171	4.0 [1.7; 7.4]	0.97 [0.34 – 2.37]
Fluvoxamine	Matched Placebo 10-day	322	16.4 [12.5; 20.6]	
	Non-matched Placebo 1-day	89	12.1 [6.3; 19.5]	0.64 [0.29 – 1.32]
	Non-matched Placebo 3-day	319	16.5 [12.7; 20.8]	0.97 [0.63 – 1.50]
	Non-matched Placebo 14-day	23	20.0 [7.1; 37.4]	1.12 [0.30 – 3.34]
	Non-matched Placebo combined	431	15.5 [12.2; 19.0]	0.92 [0.61 – 1.39]
Famotidine	Matched Placebo 10-day	264	1.5 [0.4; 3.3]	
	Non-matched Placebo 14-day	274	1.4 [0.4; 3.2]	0.96 [0.17 – 5.30]
	Non-matched Placebo combined	274	1.4 [0.4; 3.2]	0.96 [0.17 – 5.30]
Spirulina	Matched Placebo 14-day	292	1.7 [0.6; 3.5]	
	Non-matched Placebo 10-day	265	1.5 [0.4; 3.3]	0.81 [0.16 – 3.79]
	Non-matched Placebo combined	265	1.5 [0.4; 3.3]	0.81 [0.16 – 3.79]

Unadjusted Event Rate: event rates with 95% credible intervals for each category. Covariate Adjusted OR: odds ratio of non-matched placebos vs matched for each category adjusted with respect to age, sex and BMI

© 2024 Reis G et al. *JAMA Network Open.*
