# Supplementary material for: Matched vs Nonmatched Placebos in a Randomized Trial of COVID-19 Treatments
Source: JAMA Netw Open. 2024 May 20;7(5):e2410335. doi: 10.1001/jamanetworkopen.2024.10335 (PMC11107303; doi:10.1001/jamanetworkopen.2024.10335)
Supplement: Supplement 2. — Data Sharing Statement [file jamanetwopen-e2410335-s002.pdf]

Data Sharing Statement

Reis. Matched vs Nonmatched Placebos in a Randomized Trial of COVID-19 Treatments. *JAMA Netw Open*. Published May 20, 2024. doi:10.1001/jamanetworkopen.2024.10335

Data
Data available: Yes
Data types: Deidentified participant data, Data dictionary
How to access data: By email request to the Principal Investigators: Dr. Edward Mills or Dr. Gilmar Reis
When available: With publication

Supporting Documents
Document types: None

Additional Information
Who can access the data: Data requests will be considered from qualified parties at the discretion of the Principal Investigators
Types of analyses: For a specific proposed research analysis
Mechanisms of data availability: After approval of a signed data access agreement
